# The PI3K∂-Selective Inhibitor Idelalisib Induces T- and NK-Cell Dysfunction Independently of B-Cell Malignancy-Associated Immunosuppression

**DOI:** 10.3389/fimmu.2021.608625

**Published:** 2021-03-15

**Authors:** Lisa Rohrbacher, Bettina Brauchle, Ana Ogrinc Wagner, Michael von Bergwelt-Baildon, Veit L. Bücklein, Marion Subklewe

**Affiliations:** ^1^Laboratory for Translational Cancer Immunology, Gene Center, Ludwig-Maximilians-Universität München, Munich, Germany; ^2^Department of Internal Medicine III, University Hospital, Ludwig-Maximilians-Universität München, Munich, Germany; ^3^German Cancer Research Center (DKFZ), German Cancer Consortium (DKTK), Heidelberg, Germany

**Keywords:** cancer immunotherapy, chronic lymphocytic leukemia, idelalisib, immune effector cells, PI3K inhibition

## Abstract

B-cell receptors, multiple receptor tyrosine kinases, and downstream effectors are constitutively active in chronic lymphocytic leukemia (CLL) B cells. Activation of these pathways results in resistance to apoptosis and enhanced survival of the leukemic cells. Idelalisib is a highly selective inhibitor of the PI3K p110∂ isoform and is approved for the treatment of CLL in patients with relapsed/refractory disease or in those harboring 17p deletions or tp53 mutations. Despite the initial excitement centered around high response rates in clinical trials of idelalisib, its therapeutic success has been hindered by the incidence of severe opportunistic infections. To examine the potential contribution of idelalisib to the increased risk of infection, we investigated the effects of idelalisib on the immune cell compartments of healthy donors (HDs) and CLL patients. PI3K∂ blockade by idelalisib reduced the expression levels of inhibitory checkpoint molecules in T cells isolated from both HDs and CLL patients. In addition, the presence of idelalisib in cultures significantly decreased T-cell-mediated cytotoxicity and granzyme B secretion, as well as cytokine secretion levels in both cohorts. Furthermore, idelalisib reduced the proliferation and cytotoxicity of HD NK cells. Collectively, our data demonstrate that both human T and NK cells are highly sensitive to PI3K∂ inhibition. Idelalisib interfered with the functions of T and NK cell cells from both HDs and CLL patients. Therefore, idelalisib might contribute to an increased risk of infections regardless of the underlying B-cell malignancy.

## Introduction

Chronic lymphocytic leukemia (CLL) is characterized by impairment of the immune system and is therefore associated with an increased susceptibility to opportunistic infections (1–5). Several factors contribute to this increased risk profile: CLL cells compromise the development of healthy B cells, cause immunosuppression due to their close proximity to effector cells, modulate T-cell function, and cause immunoglobulin deficiency (6–9). Therefore, immunotherapeutic approaches are indicated and first-line treatments consist of rituximab or obinutuzumab as part of either chemoimmunotherapy or targeted therapy with ibrutinib and venetoclax (10). The latter target B-cell receptor (BCR) signaling and downstream receptor tyrosine kinases, which play a key role in the pathogenesis of CLL (11–17).

Idelalisib is a potent small-molecule inhibitor of phosphoinositide 3-kinases (18, 19). PI3K is one of the most commonly activated kinases in the BCR signaling cascade (20–23). Class I PI3Ks are comprised of a regulatory subunit and one of four catalytic subunits (p110 α, β, γ, and ∂)(24–27). These isoforms differ in tissue expression: PI3Kα and PI3Kβ are ubiquitously expressed, whereas PI3Kγ and PI3K∂ are highly enriched in the hematopoietic compartment (19, 21, 25, 28–30). Mechanistically, PI3K∂ activates the serine/threonine kinases AKT and mammalian target of rapamycin (mTOR), which leads to proliferation, differentiation, and enhanced survival of the cancer cells (19, 24, 25, 31–33). Idelalisib binds to the ATP-binding pocket of the catalytic subunit of PI3K, thereby specifically abrogating downstream PI3K∂/AKT signaling and inducing apoptosis of malignant cells (34, 35). Idelalisib, has been evaluated in conjunction with rituximab in a randomized, double-blind, phase III study in patients with CLL (ClinicalTrials.gov Identifier: NCT01539512). Due to high response rates of 81%, idelalisib was approved by the US Food and Drug Administration for the first-line treatment of CLL patients with the 17p deletion or TP53 mutation, and also in the relapsed or refractory (r/r) setting (35, 36).

However, after the initial excitement, three clinical trials involving idelalisib reported a high rate of adverse events, including severe diarrhea, liver toxicity, pneumonitis, severe colitis, and serious infections (37–42) (ClinicalTrials.gov Identifiers: NCT01539512, NCT01732913, NCT01569295). Although a high rate of opportunistic infections in CLL patients is well-documented (43–45), idelalisib treatment not only increased the incidence but also added other immune-related adverse events (46). In a randomized phase III trial in r/r CLL, patients were treated with Rituximab plus Idelalisib (IDELA/R-to-IDELA) vs. Rituximab monotherapy (placebo/R). The group with IDELA/R-to-IDELA had a higher incidence of infection or infestation: 53.6 vs. 23.1%, with lower respiratory tract infection in 23.6 vs. 11.1% (47). Opportunistic infections were a common cause with 5 vs. 1 patient presenting with pneumocystis jirovecii pneumonia, 2 vs. 0 patient presenting with cytomegaly virus infection and 22 patients with fungal infection in the IDELA/R-to-IDELA group. Confirmatory data were reported from another phase III trial comparing ofatumumab with and without Idelalisib in pretreated CLL patients with serious infections being more common in the ofatumumab plus idelalisib group: pneumonia was reported in 23 vs. 8 patients, sepsis in 11 vs. 1 patient, and pneumocystis jirovecii pneumonia in 8 vs. 1 patient (48). This resulted in 22-treatment-related deaths in the ofatumumab plus Idelalisib vs. only 6-treatment-related deaths in the ofatumumab group.

These observations suggest for an additive negative impact of idelalisib on immune effector cells. Although many studies have focused on the influence of idelalisib on CLL cells, less is known about the impact of PI3K∂ blockade on healthy immune cells.

In this study, we investigated the immunomodulatory influence of idelalisib on the adaptive cellular immune compartment. To start dissecting the effect of idelalisib on cellular immune responses independently of its impact on CLL cells, we isolated T and natural killer (NK) cells from healthy donor (HD) peripheral blood (PB). We analyzed changes in the proliferative behavior, cytokine secretion, and cytotoxic capacity of cocultures in the presence of idelalisib. In a second step, we sought to replicate these findings with samples from CLL patients. Our data suggest that idelalisib interferes with T- and NK-cell function, thereby adding to the already increased rate of opportunistic infections in CLL patients.

## Materials and Methods

### Idelalisib and Key Reagents

Idelalisib was provided by Gilead Sciences. A 10 mM stock solution of idelalisib was prepared in dimethyl sulfoxide (DMSO; Serva, 20385.01) and stored at −20°C. The used concentrations of 0.05, 0.5, and 1 μM were chosen to mimic the peak plasma concentrations observed in patients after 150 mg twice daily administration of idelalisib (49). Sources of all key reagents are listed in Supplementary Table 1.

### Patients

PB samples from HDs and patients with CLL were collected with written consent in accordance with the Declaration of Helsinki and approval by the Institutional Review Board of the Ludwig-Maximilian University of Munich. Patient characteristics are summarized in Table 1. The patients had a median age of 64.5 years with a percentage of female patients of 46.7%.

**Table 1:** Patient characteristics.

**PT**	**Gender**	**Age**	**RAI stage**	**Binet stage**	**TP53 mutational status**	**Del(17p13)**	**IgVH mutational status**	**Del(13q14)**
1	M	64	III	B	–	–		
2	M	74	III	C	–	–		+
3	M	55	III	C	–	–		
4	F	66	0	A			+	+
5	M	48	I	A			+	+
6	F	81	I	A				–
7	F	59	I	A			+	+
8	M	60	II	B	–	–	+	+
9	F	59	III	C	–	–		+
10	M	82	II	B	–	–		–
11	F	69	I	B	–	–		–
12	F	67	III	C	–	–		–
13	M	73	IV	C	–	–		–
14	-	80						
15	M	75	III	C	–	–		–

*+, positive; –, negative; blank, not determined*.

### Cell Lines

Cell lines were obtained from the German Collection of Microorganisms and Cell Cultures (DSMZ, Braunschweig, Germany) and were authenticated by their short tandem repeat profile. All cell lines were tested monthly for *Mycoplasma* contamination with the MycoAlert Mycoplasma Detection Kit (Lonza, LT07-705) according to the manufacturer's instructions. Cells were passaged twice a week and cultured in RPMI 1640 medium (PAN Biotech, P04-16500) media supplemented with 10% fetal calf serum (Thermo Fisher Scientific, 10270106), 1% HEPES (Carl Roth, HN78.1) and 1% penicillin-streptomycin-glutamine (PSG) (Thermo Fisher Scientific, 10378016) at 37°C in a 5% CO_2_ atmosphere. Cells were used within 2 months of thawing.

### Source of Primary Cells

HDs had a median age of 28.4 years with a percentage of females of 48.3%. To overcome the age discrepancy, we extended the trial on an elderly HD cohort (*n* = 4, Supplementary Figure 2). The median age of this cohort was 62 years with a percentage of 50% females. PB mononuclear cells (PBMCs) from HD were isolated by density gradient centrifugation (using Biochrom separating solution, L6115) from PB and either cryopreserved at < -80°C in cell culture medium (described above) containing 10% DMSO, or directly used for experiments. T cells were negatively isolated from frozen HD PBMCs with the human Pan T Cell Isolation Kit (Miltenyi Biotec, 130-096-535) and cultured in cell culture medium. NK cells were either isolated negatively with the human NK Cell Isolation Kit (Miltenyi Biotec, 130-092-657) or with the EasySep Human CD56 Positive Selection Kit II (Stemcell Technologies, 17815) from fresh HD PBMCs and cultured in NK MACS Medium (Miltenyi Biotec, 130-114-429) supplemented with 5% human serum (Sigma–Aldrich, H6914). Monocytes were positively isolated from fresh HD PBMCs with human CD14 Microbeads (Miltenyi Biotec, 130-050-201). Neutrophils were negatively isolated from fresh HD PB with the EasySep Direct Human Neutrophil Isolation Kit (Stemcell Technologies, 19666).

HD-derived immune cells were cultured with 0.05, 0.5, or 1 μM idelalisib or with the DMSO concentration that matches the DMSO concentration of the drug-treated cultures as vehicle controls.

### Flow Cytometry

All measurements were conducted on a CytoFLEX flow cytometer (Beckman Coulter) and analyzed using FlowJo software (BD Biosciences, version 10; RRID: SCR_008520). All antibodies used in the following experiments are listed in Supplementary Table 1. Median fluorescence intensity (MFI) was determined, and the MFI ratio (MFI sample/MFI isotype control) was calculated (Supplementary Figure 1).

### *In vitro* Cell Proliferation Assay

HD T cells were stained using the CellTrace CFSE Proliferation Kit (Thermo Fisher Scientific, C34554) according to the manufacturer's instructions. For stimulation, CD3/CD28 Dynabeads (Thermo Fisher Scientific, 11131D) at a bead-to-cell ratio of 1:2 and 30 U/mL interleukin-2 (IL-2; R&D Systems, 202-IL-010/CF) were added to the culture for 5 days. T cell subsets were discriminated by the expression of the chemokine receptor CCR7 in combination with the naïve cell marker CD45Ra (Supplementary Figure 2). NK cells were stimulated by addition of 500 U/mL IL-2 for 10 days. Fold change was calculated as: “Viable NK cell count day 10”/“Viable NK cell count day 0.”

### Cytometric Bead Array

HD T cells were stimulated as described above. After 3 days, the secretion of interferon-γ (IFN-γ), tumor necrosis factor (TNF), IL-2 and interleukin 10 (IL-10) was measured by analyzing the cell culture supernatant in a Th1/Th2 cytometric bead array (BD Biosciences, 551809) (50, 51). The assay was performed according to the manufacturer's instructions using flow cytometry.

### T-Cell Cytotoxicity Assay

The cytotoxic capacity of T cells was assessed in two different assays. In the first assay, HD T cells were activated as described above. After 3 days the secretion of the cytolytic molecules perforin and granzyme B was analyzed by multiparameter flow cytometry. In the second assay, the T-cell recruiting antibody-mediated lysis of the target cell line HL-60 at an effector– to–target ratio (E:T) of 1:3 was measured. Either an anti-CD33 bispecific T-cell-recruiting antibody (5 ng/mL) (52), recognizing CD3 on T cells and CD33 on target cells, or no antibody was added to the coculture. After 72 h, target and T cell counts were analyzed by flow cytometry. Lysis was calculated according to the formula %lysis = 100 – [#target cells (with antibody)]/[#target cells (without antibody)]^*^100.

### NK-Cell Cytotoxicity Assay

The cytotoxic capacity of NK cells was assessed in two different assays. In the first assay, the cell line K562 was stained with calcein AM (Thermo Fisher Scientific, C3100MP) according to the manufacturer's instructions. Freshly isolated HD NK cells were cocultured with the stained K562 cells at an E:T ratio of 10:1 in presence of idelalisib or DMSO. After 4 h, the fluorescence intensity of the co-culture supernatant was measured on a microplate reader (excitation: 485 nm; emission: 535 nm). Lysis was calculated according to the formula [(*F*_test_) – (*F*_spontaneous_)/(*F*_maximum_) – (*F*_spontaneous_)] × 100, where *F*_spontaneous_ represents the fluorescence intensity of calcein released from target cells in medium alone, and *F*_maximum_ is the fluorescence intensity of calcein released from target cells lysed in medium containing 2% Triton X-100 (Sigma–Aldrich, X100-5ML), each measured in at least three replicate wells. In the second assay, freshly isolated HD NK cells were cocultured with K562 or Jurkat cells at an E:T ratio of 5:1 for 20 h in presence of idelalisib or DMSO. After 20 h, specific lysis was analyzed by multiparameter flow cytometry (Beckman Coulter CytoFLEX S flow cytometer). The percentage of lysis was determined as the target cell count of idelalisib-treated relative to the control cultures.

### Phagocytosis Assay

Monocyte function was assessed by measuring phagocytosis of pHrodo Green *E.coli* BioParticles (Thermo Fisher Scientific, P35366). Freshly isolated monocytes were incubated with the pHrodo particles for 2 h, then phagocytosis was analyzed by flow cytometry.

### Neutrophil Activation Assay

Neutrophil function was analyzed over 4 h in a standard Seahorse XF neutrophil activation assay according to the manufacturer's instructions.

### Statistics

Statistical analyses were performed using GraphPad Prism Software Version 8.4.2. As statistical test to compare the two treatment groups Wilcoxon matched signed-rank test was used. *P-*values and the number of replicates performed to derive the data are indicated in the figure legends.

## Results

### Inhibition of the PI3K∂ by Idelalisib Reduces the Expression Levels of Inhibitory Checkpoint Molecules in CD3^+^ T Cells and Treg Cells

**Figure 1:**
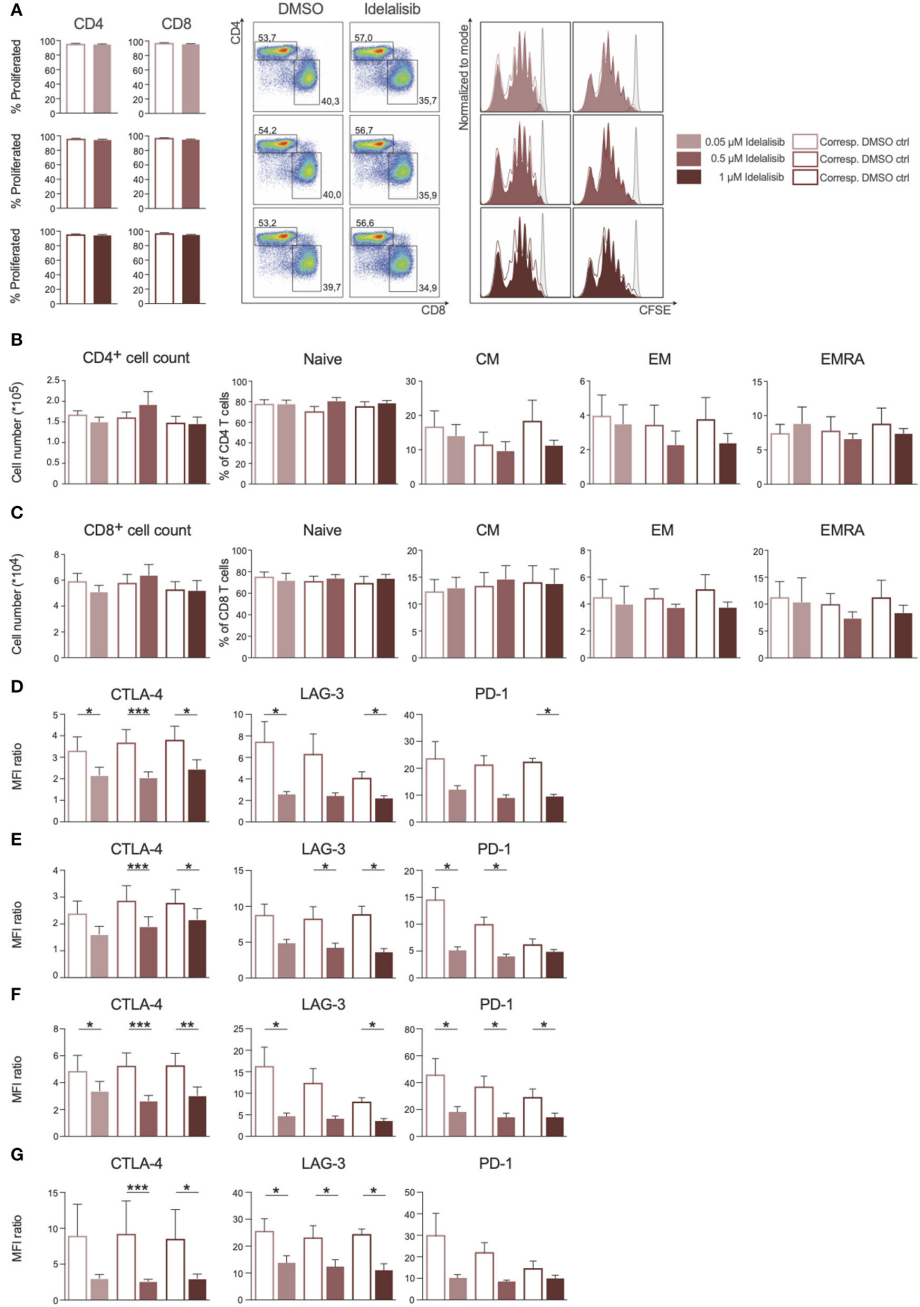
Inhibition of the PI3K∂ signaling pathway by Idelalisib reduces the expression levels of inhibitory checkpoint molecules in CD3^+^ T cells and Treg cells. **(A)** Compiled data showing the percentage of proliferated CD4^+^ and CD8^+^ T cells in the presence of various concentrations of idelalisib or DMSO, and representative flow cytometry dot plots and histograms. **(B,C)** Bar charts depicting the development of CD4^+^ and CD8^+^ T-cell subsets: naïve, central memory (CM), effector memory (EM), and terminally differentiated effector memory cells re-expressing CD45RA (ERMA) after 6 days of coculture, with different concentrations of idelalisib or DMSO as vehicle control. **(D–G)** Bar graphs of MFI ratios showing the expression levels of checkpoint molecules CTLA-4, LAG-3, and PD-1 in CD4^+^ T cells, CD8^+^ T cells, CD4^+^ Tregs and CD8^+^ Tregs after stimulation for 3 or 5 days with IL-2 and CD3/CD28 activation beads. Error bars represent mean ± SEM; *p ≤ 0.05, **p ≤ 0.005, ***p ≤ 0.0005; *n* = 6–12 HDs.

As PI3K∂ was previously shown to be important for T-cell signaling, we evaluated the effect of PI3K∂ blockade on T-cell proliferation. We found that proliferation of CD4^+^ and CD8^+^ T cells (Figure 1A) and the respective subsets (Figures 1B,C) was not affected by idelalisib. Next, we analyzed the expression levels of several inhibitory checkpoint molecules in T cells and Tregs after stimulation for 72 or 120 h in the presence of idelalisib. The expression of cytotoxic T-lymphocyte-associated protein 4 (CTLA-4), lymphocyte activation gene 3 (LAG-3), and programmed cell death protein 1 (PD-1) was significantly downregulated in idelalisib-treated T cells and Tregs in comparison to the DMSO vehicle control cells (Figures 1D–G). Our results demonstrate that proliferation of T cells is not solely dependent on signaling through PI3K∂ and thus is not susceptible to inhibition of PI3K∂ by idelalisib. Furthermore, our data demonstrate that blockade of PI3K∂ signaling reduced expression of inhibitory checkpoint molecules in T cells and Tregs.

### Inhibition of PI3K∂ by Idelalisib Leads to Decreased T-Cell-Mediated Cytotoxicity Against Target Tumor Cells and Reduced Secretion of IL-10, TNF, and IFN-γ

**Figure 2:**
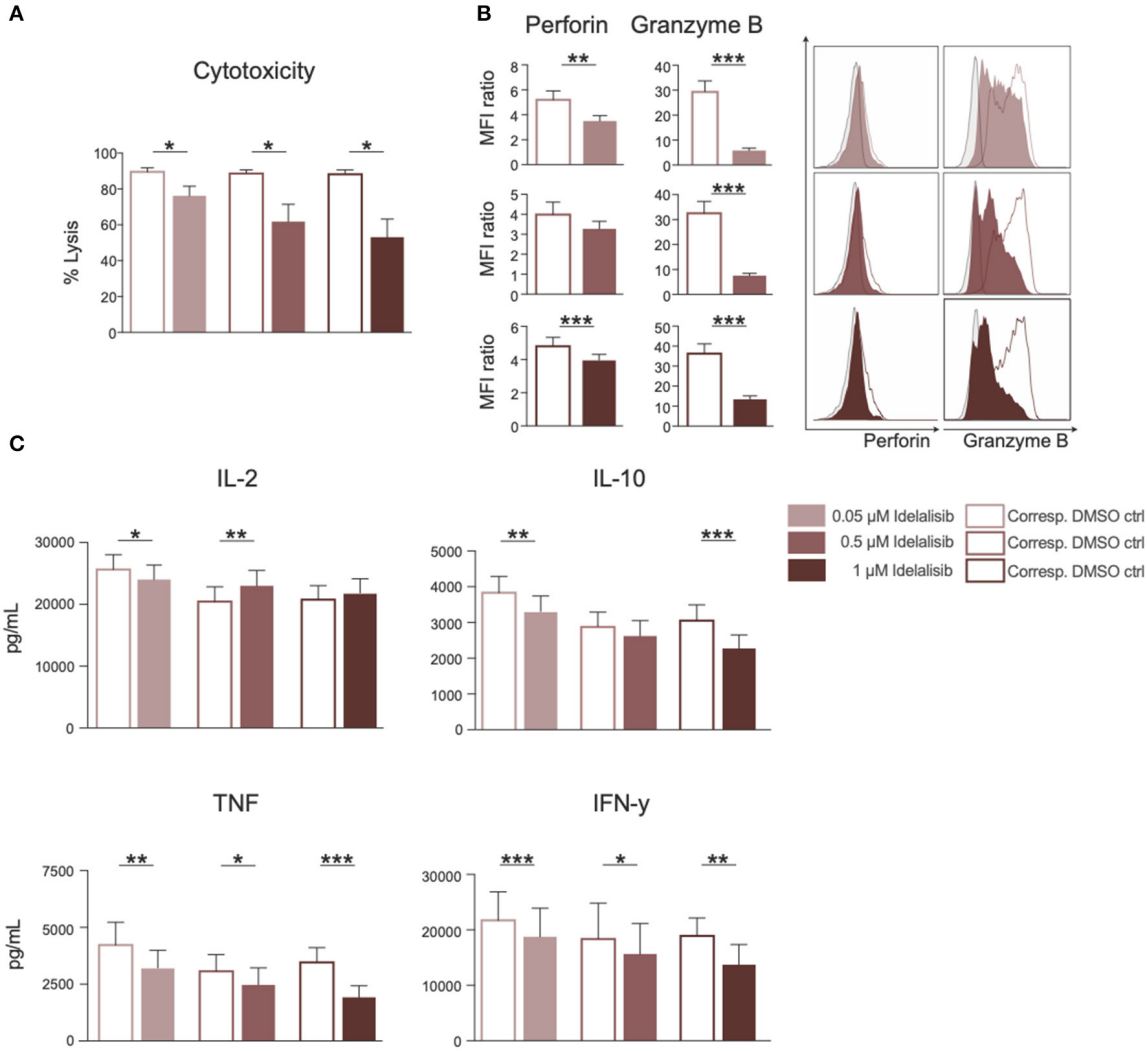
Inhibition of PI3K∂ by idelalisib leads to decreased T-cell-mediated cytotoxicity against target tumor cells and reduced secretion of IL-10, TNF, and IFNγ. **(A)** Cytolytic capacity of CD3^+^ T cells in coculture with target HL-60 cell line and various concentrations of idelalisib after 72 h. **(B)** Bar charts and representative overlaid flow cytometric histograms depicting expression levels of perforin and granzyme B in CD8 T cells after bead mediated activation for 72 h in the presence of different concentrations of idelalisib or DMSO. **(C)** Bar charts representing the levels of IL-2, IL-10, TNF, and IFNγ in the supernatant of the coculture after 72 h. Error bars represent mean ± SEM; *p ≤ 0.05, **p ≤ 0.005, ***p ≤ 0.0005, *n* = 12 HDs.

To test whether the downregulation of inhibitory checkpoint molecules in T cells also mirrors reduced T-cell effector function, we analyzed the effect of PI3K∂ blockade on T-cell cytotoxicity and cytokine secretion. The cytotoxicity of T cells against target HL−60 cells was significantly reduced in a coculture in the presence of idelalisib (Figure 2A). To further investigate if this reduction in the cytolytic capacity was due to a decrease in secretion of cytolytic molecules, T cells were analyzed for perforin and granzyme B secretion after 72 h stimulation in the presence of idelalisib. There was a significantly lower degranulation of perforin and granzyme B in the cultures containing idelalisib (Figure 2B). Next, we evaluated the effect of PI3K∂ blockade on the secretion of different cytokines after stimulation for 72 h. Secretion levels of IL-10, TNF, and IFN-γ were significantly reduced in the presence of idelalisib. In contrast, secretion of IL-2 was slightly increased (Figure 2C). Taken together, these results demonstrate that blockade of PI3K∂ signaling with idelalisib has a negative impact on the effector functions of T cells, such as cytotoxicity and cytokine secretion.

### Inhibition of PI3K∂ by Idelalisib Reduces NK-Cell Proliferation and the Percentage of Cytotoxic NK Cells

**Figure 3:**
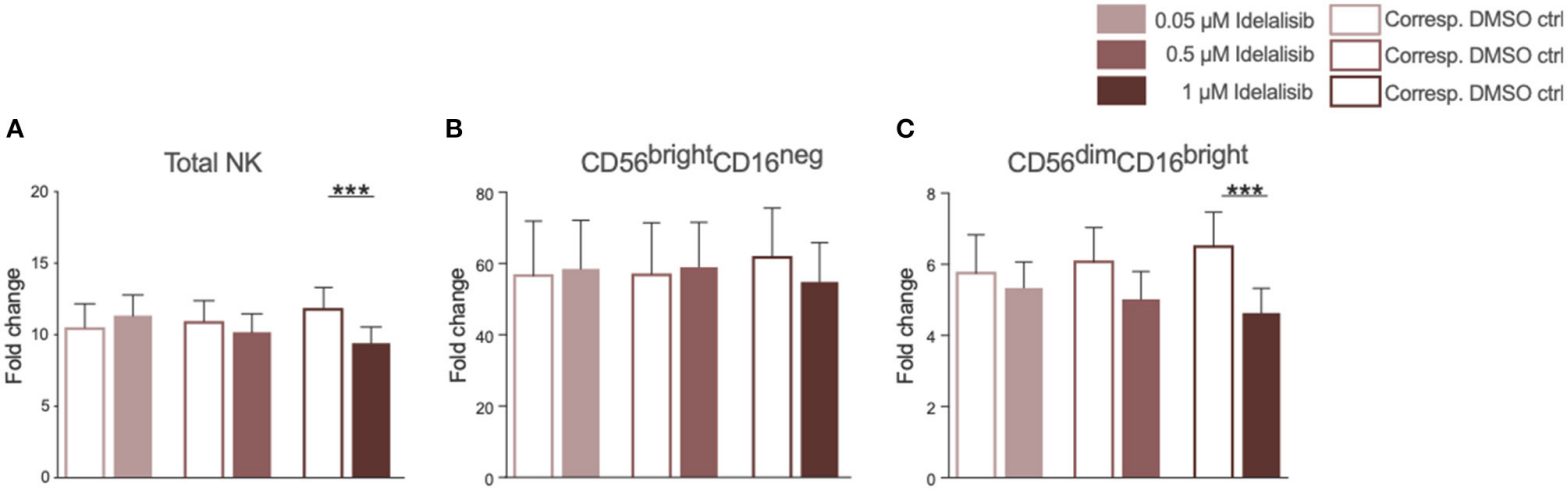
Inhibition of PI3K∂ by idelalisib reduces NK-cell proliferation and percentage of cytotoxic NK cells. **(A)** Compiled bar chart showing the proliferative capacity of total CD3^−^CD56^+^ NK cells after 10 days in the presence of IL-2 and different concentrations of Idelalisib or DMSO as vehicle control. **(B,C)** The shift in the NK-cell composition is depicted as the fold change between days 0 and day 10 of the subsets CD56^bright^CD16^neg^ and CD56^dim^CD16^bright^. Error bars represent mean ± SEM; ***p ≤ 0.0005; *n* = 12 HDs.

As we found that inhibition of PI3K∂ significantly impairs important functions of the T-cell compartment, we sought to evaluate if similar effects can be seen in NK cells, the cytotoxic lymphocytes of the innate immune system. The addition of idelalisib to IL-2 stimulated NK cells led to a decreased NK-cell expansion in comparison to the DMSO-treated control (Figure 3A). Furthermore, idelalisib affected the proliferation of the cytotoxic NK-cell population over the 10-day period. As the percentage of dead cells in both conditions is not significantly different, this observation is most likely due to a reduced proliferation instead of increased apoptosis in the idelalisib treated condition (media %dead cells 1 μM idelalisib vs. corresponding DMSO control: 24.3 vs. 25.5, *n* = 12, data not shown). The cytokine producing CD56^bright^CD16^neg^ compartment showed no differences between cultures (Figure 3B). However, the proliferative capacity of the cytotoxic CD56^dim^CD16^bright^ subset was reduced upon PI3K∂ blockade (Figure 3C).

### Inhibition of PI3K∂ by Idelalisib Decreases NK-Cell Cytotoxicity

**Figure 4:**
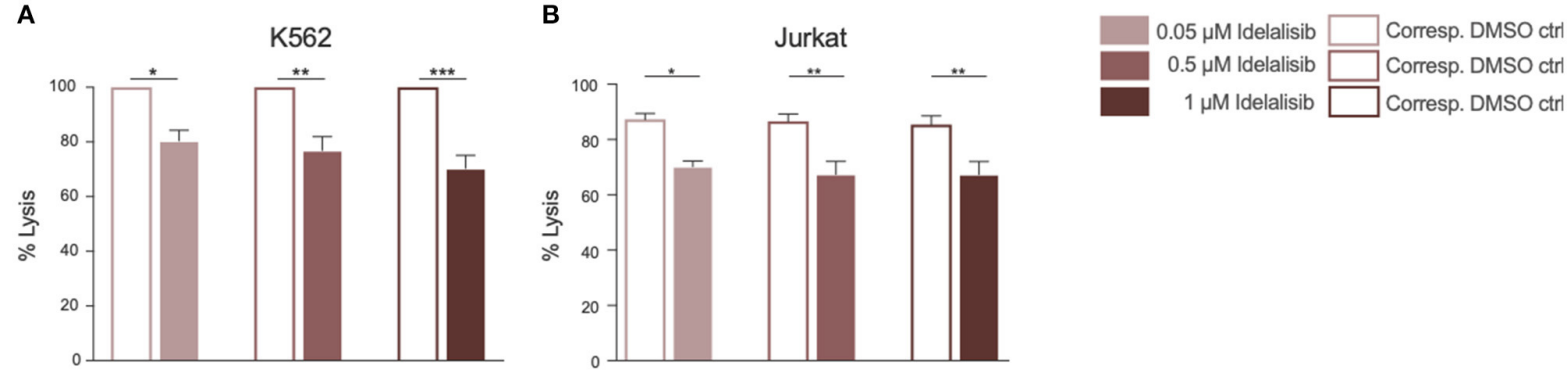
Inhibition of PI3K∂ by idelalisib decreases NK-cell cytotoxicity. **(A,B)** Cytotoxicity of CD3^neg^CD56^pos^ NK cells against the K562 cell line in a standard calcein-release assay and against the Jurkat cell line in a flow cytometry-based coculture assay. Error bars represent mean ± SEM; *p ≤ 0.05, **p ≤ 0.005; *n* = 12 HDs.

To evaluate if this reduction translates into impaired cytotoxicity, we analyzed the cytolytic capacity of NK cells toward target cells in presence of idelalisib. NK cells are capable of killing target cells via two different apoptotic pathways, either through perforin- and granzyme- mediated lysis or through death receptor ligation with, for example, the Fas ligand (FasL). To investigate if idelalisib interferes with both apoptotic pathways, we separately cocultured two target cell lines with HD NK cells. The cytotoxicity of NK cells toward target K562 cells, which are lacking the MHC class I antigen and are thus killed via granzyme B-mediated lysis, was significantly reduced (Figure 4A). Lysis of target Jurkat cells, which are killed in a Fas–FasL-dependent manner, was also significantly decreased in the presence of idelalisib (Figure 4B). Together our data show that PI3K∂ signaling is important for NK cell-mediated lysis of cancer cells *in vitro* and that blockade by idelalisib significantly reduces the cytolytic capability of NK cells.

### Inhibition of PI3K∂ by Idelalisib Alters Neither the Phagocytic Capacity of Monocytes Nor the Activation of Neutrophils

**Figure 5:**
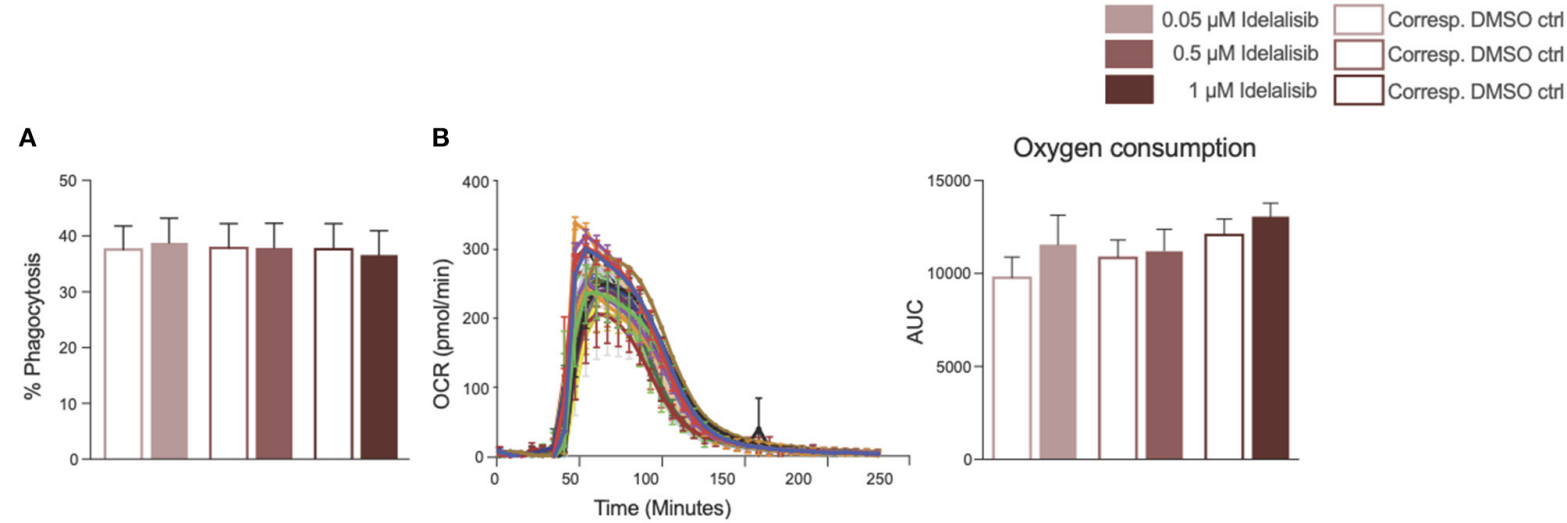
Inhibition of PI3K∂ by idelalisib alters neither monocyte nor neutrophil function. **(A)** Compiled data showing the phagocytic capacity of monocytes after 4 h in the presence of different concentrations of idelalisib or DMSO as vehicle control; *n* = 12 HDs. **(B)** Representative kinetic trace of the oxygen consumption rate (OCR) and compiled bar chart depicting the oxidative burst of neutrophils as areas under the curve (AUC) in a Seahorse XF neutrophil activation assay; *n* = 8 HDs.

To further investigate the impact of idelalisib on other innate immune cells, we studied the effect of PI3K∂ blockade on the phagocytic capacity of monocytes and neutrophil activation in two independent short-term assays. No differences in the phagocytosis rates of monocytes were detected (Figure 5A). Neutrophil activation was measured via the generation of reactive oxygen species, termed an “oxidative burst,” in a Seahorse XF neutrophil activation assay. Neutrophils in the idelalisib-treated group did not show a significant difference in oxygen consumption rates in comparison to the vehicle-treated group (Figure 5B). These data indicate that PI3K∂ blockade does not have a direct impact on monocyte phagocytic capacity and neutrophil activation in these short-term assessments.

### Inhibition of PI3K∂ by Idelalisib Has a Negative Impact on Effector Functions of T Cells Derived From CLL Patients at the Time of Initial Diagnosis

**Figure 6:**
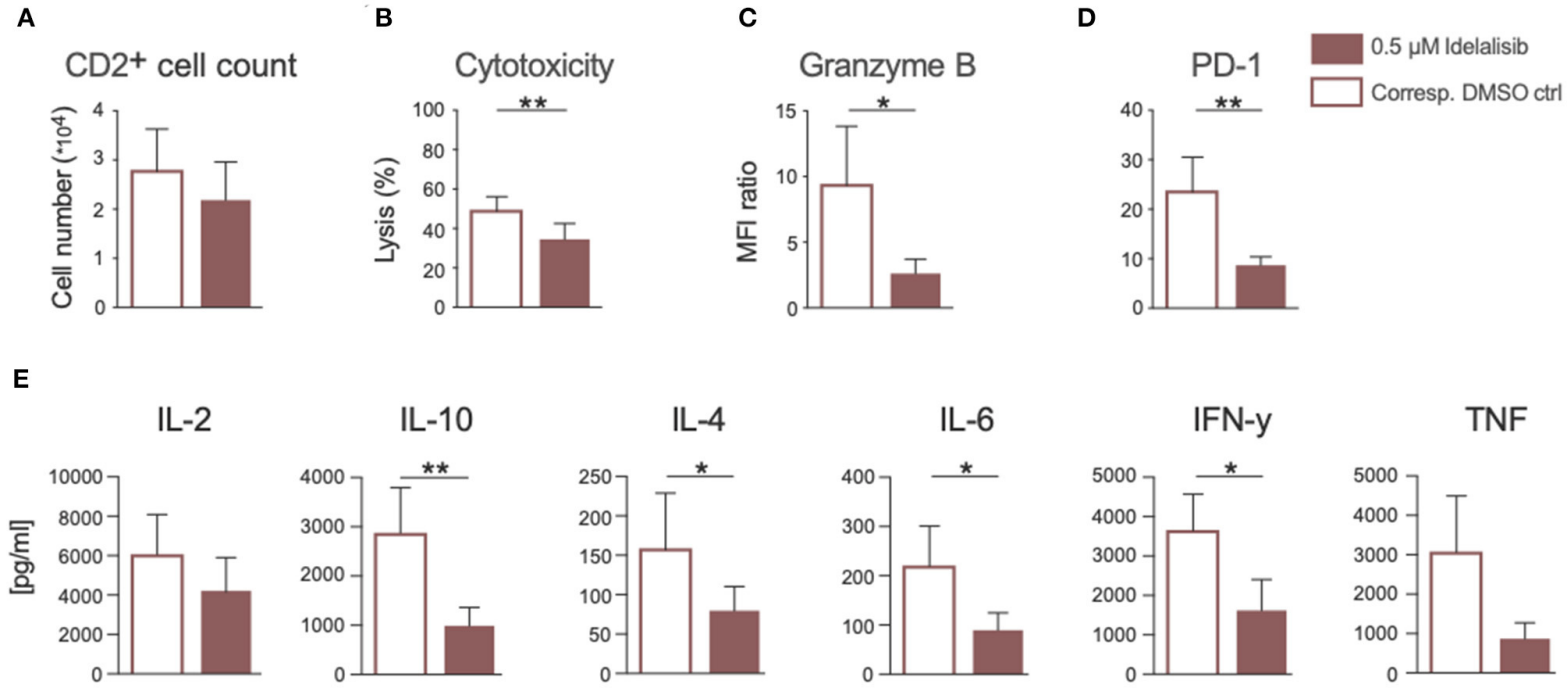
Inhibition of PI3K∂ by idelalisib impairs cytotoxicity and cytokine secretion of T cells isolated from CLL patient samples. **(A)** Compiled data showing the percentage of proliferated CD2^+^ T cells in the presence or absence of idelalisib (*n* = 15). **(B)** Cytolytic capacity of CD3^+^ T cells in coculture with target HL−60 cell line and idelalisib after 72 h. **(C,D)** Bar charts depicting expression levels of granzyme B and PD-1 in T cells after 72 h coculture with HL-60 cells in the presence of idelalisib or DMSO (*n* = 6). **(E)** Bar charts representing the levels of IL-2, IL-10, IL-4, IL-6, TNF, and IFNγ in the supernatant of the coculture after 72 h in the presence or absence of idelalisib (*n* = 15). Error bars represent mean ± SEM; *p ≤ 0.05, **p ≤ 0.005.

Next, we analyzed if the previously described effects of PI3K∂ inhibition could also be seen if CLL patient cells were treated in culture with idelalisib. To this end, we isolated T cells from cryopreserved PBMCs of CLL patients at time of initial diagnosis. Again, patient samples treated with the vehicle DMSO served as controls. PI3K∂ blockade by idelalisib did not alter the proliferative capacity of T cells isolated from CLL PBMCs (Figure 6A). The cytotoxicity of CLL T cells against target HL-60 cells was significantly reduced in a coculture treated with idelalisib (Figure 6B). This could be further verified by a significant decrease in granzyme B secretion in a culture containing the inhibitor (Figure 6C). Furthermore, PD-1 expression was significantly decreased in the idelalisib-treated T-cell group (Figure 6D). Next, we evaluated the effect of PI3K∂ blockade on the secretion of different cytokines after coculture for 72 h with the target HL-60 cell line. Cytometric bead array analysis of CLL T cells revealed significantly reduced secretion levels of IL-10, IL-4, IL-6, and IFN-γ (Figure 6E). Taken together, these results demonstrate that blockade of PI3K∂ signaling with idelalisib has a negative impact on the effector functions of CLL T cells, such as cytotoxicity and cytokine secretion. However, differences in age are possible cofounding variables.

## Discussion

PI3K∂ inhibition by idelalisib has proven to be highly effective in the treatment of r/r CLL patients. However, this clinical success is somewhat diminished by the increased rate of opportunistic infections in these patients (37–42). The factors contributing to this observation are incompletely understood.

In the present study, we evaluated the effect of PI3K∂ blockade by idelalisib on the non-malignant human immune cell compartment of healthy individuals. First, we analyzed the effects of idelalisib on T cells and Tregs. PI3K∂ blockade by idelalisib did not have an impact on the proliferative capacity of HD T cells. However, we observed a significant decrease in the expression levels of the inhibitory checkpoint molecules PD-1, CTLA-4, and LAG-3 in both T cells and Tregs in cultures containing idelalisib. Our findings are supported by previous studies, which described PI3K∂ as the main transducer of PI3K signaling in human T cells (25, 53) and Tregs (54). Next, we evaluated if these findings correlated to impaired T-cell function. We demonstrated that secretion of IL-10, TNF, and IFNγ by idelalisib-treated HD T cells was significantly reduced. This is consistent with previous studies that looked at the impact of other PI3K∂-blocking agents on cytokine secretion of T cells in mice (25, 53, 55). Furthermore, we observed that PI3K∂ blockade significantly decreased the cytolytic capacity of HD T cells. This manifested through a significantly reduced secretion of the cytolytic molecules perforin and granzyme B, as well as a significant decrease in antibody-mediated target cell killing. Together, our data show that idelalisib severely impairs the functions of T cells and Tregs isolated from HDs. Combined with the findings of a previous study by Chellappa and colleagues (56), our data support the hypothesis that idelalisib leads to T and NK cell dysfunction. Therefore, our *in vitro* observations might reflect the increased rate of opportunistic infections in treated CLL patients. Idelalisib exposure led to a significant decrease in the expression of PD-1, which is often described as a marker for T-cell activation. This might be an indicator for reduced T-cell activity and in turn a dampened immune response, which might be a factor behind the increased rates of infections measured in idelalisib-treated CLL patients.

Emerging data from clinical trials suggests that the improved T-cell-mediated antitumor response and the impressive clinical outcome in CLL might be due to reduced Treg numbers and their reduced suppressive function in idelalisib-treated patients (57, 58). Conversely, inhibiting the suppressive activity of Tregs can also lead to severe adverse autoimmune effects (56, 59).

As a next step, we wanted to evaluate the effect of idelalisib on NK cells, the cytotoxic lymphocytes of the innate immune system. Previous studies in mice with defective PI3K∂ have suggested that PI3K plays a critical role in NK-cell effector function (60, 61). Furthermore, Zebedin and colleagues demonstrated that selective inhibition of PI3K∂ in mice leads to impaired degranulation and target cell killing by NK cells (62). Therefore, we wanted to evaluate whether PI3K∂ blockade also has a negative impact on the human NK-cell compartment. In our study, we observed that idelalisib reduced NK-cell proliferation. Moreover, PI3K∂ inhibition led to a decrease in the percentage of cytotoxic NK cells, which also translated into reduced target cell killing by NK cells. We show that two different apoptosis pathways are affected. Idelalisib impaired cell death through secretion of cytolytic molecules, as well as cell death via the Fas–FasL pathway. NK cells are an important part of the innate immune system and play a key role in the defense against infections. Taken together, our data demonstrate a decrease NK-cell proliferation and cytolytic activity by idelalisib and that this might contribute to the increased frequency of infectious events observed in clinical trials.

CLL has been associated with profound defects in T-cell function and synapse formation (63–66). These T-cell defects are thought to result in failure of antitumor immunity and increased susceptibility to infections (67, 68). In view of our findings on healthy T cells, PI3K∂ inhibition might have an even more pronounced effect on the immune response of CLL patients, contributing to an elevated risk of severe side effects such as opportunistic infections. To confirm the clinical relevance of our findings and that these effects might indeed contribute to the increased rate of infections during idelalisib therapy, we isolated T cells from cryopreserved PBMCs collected from CLL patients at the time of initial diagnosis. As expected, the proliferative capacity of these T cells was lower compared to healthy T cells. Idelalisib had only a minor impact on the proliferation on T cells from CLL patient. However, we observed a significant decrease in the cytolytic capacity of CLL T cells treated with idelalisib. This was supported by our finding of a significantly reduced level of secreted granzyme B. Consistent with our data from HDs, PD-1 expression in T cells was significantly decreased in the presence of idelalisib, most likely as a result of reduced T-cell activation. In line with these findings, we also observed a significantly reduced secretion of IL-10, IL-4, IL-6, and IFN-γ; secretion of IL-2 and TNF also appeared to be affected, albeit to a lesser extent. As proinflammatory cytokines are mainly secreted by Tregs, the decrease in IL-10 secretion might indicate impaired Treg suppressive function, which could also contribute to a higher risk of autoimmune diseases. Furthermore, reduced TNF and IFNγ secretion serves as an indicator of less activated, less functional, or even exhausted T cells.

Our data demonstrates that both human T and NK cells are highly sensitive to PI3K∂ inhibition. Idelalisib interfered with the functions of T and NK cells from both HDs and CLL patients. In summary our *in vitro* data suggest that idelalisib-induced impairment of T and NK-cell function contributes to an increased rate of infections regardless of the underlying B-cell malignancy.

## Data Availability Statement

The original contributions presented in the study are included in the article/Supplementary Material, further inquiries can be directed to the corresponding author.

## Ethics Statement

The studies involving human participants were reviewed and approved by PB samples from HDs and patients with CLL were collected with written consent in accordance with the Declaration of Helsinki and approval by the Institutional Review Board of the Ludwig-Maximilian University of Munich. The patients/participants provided their written informed consent to participate in this study.

## Author Contributions

MS, VB, and MB-B: conceptualization and funding acquisition. LR, BB, and AO: methodology. LR, BB, and AO: investigation. LR: writing—original draft. AO, BB, VB, and MS: writing, review, and editing. LR, BB, and MS: review. MS: supervision. All authors read and approved the final manuscript.

## Conflict of Interest

The authors declare that the research was conducted in the absence of any commercial or financial relationships that could be construed as a potential conflict of interest.

## Supplementary Material

Supplementary Figure 1 Descriptive Plots. **(A)** Flow cytometry histogram: calculation of the MFI ratio (Median fluorescence intensity ratio). **(B)** Gating strategy T-cell subset discrimination. The four subsets were discriminated as follows: naïve T cells: CD45Ra^+^/CCR7^+^, memory T cells: CD45Ra^−^/CCR7^+^, effector T cells: CD45Ra^−^/CCR7^−^, and terminally differentiated effector memory T cells: CD45Ra^+^/CCR7^−^. **(C)** Representative flow cytometry histograms, MFI ratio: CTLA-4 (idelalisib = 1.9; DMSO = 4.7), LAG-3 (MFI ratios: idelalisib =2.3; DMSO = 19.5), and PD-1 (MFI-ratios: idelalisib = 13.7; DMSO = 39.3).

Supplementary Figure 2 Inhibition of PI3K∂ by idelalisib in an older reference cohort. **(A)** Compiled data showing the percentage of proliferated CD4^+^ and CD8^+^ T cells in the presence of various concentrations of idelalisib or DMSO. **(B–E)** Bar graphs of median fluorescence intensity (MFI) ratios showing the expression levels of checkpoint molecules CTLA-4, LAG-3, and PD-1 in CD4^+^ T cells, CD8^+^ T cells, CD4^+^ Tregs, and CD8^+^ Tregs after stimulation for 3 days with IL-2 and CD3/CD28 activation beads. **(F)** Cytolytic capacity of CD3^+^ T cells in coculture with HL-60 cell line and various concentrations of idelalisib after 72 h. **(G)** Bar charts depicting expression levels of perforin and granzyme B in CD8^+^ T cells after stimulation with CD3/CD28 beads for 72 h in the presence of various concentrations of idelalisib or DMSO. **(H)** Cytotoxicity of CD3^−^CD56^+^ NK cells against the K562 cell line in a flow cytometry-based coculture assay. Error bars represent ± SEM, *n* = 4 HDs in all depicted assays.

Supplementary Table 1 Key Resources.

## Abbreviations

PI3K∂: Phosphoinositide 3-kinase delta
PB: Peripheral blood
PBMC: Peripheral blood mononuclear cell
CLL: Chronic lymphocytic leukemia
HD: Healthy donor
NK cell: Natural killer cells
AKT: Protein kinase B, or PKB
mTOR: mammalian target of rapamycin
CTLA-4: Cytotoxic T-lymphocyte-associated protein 4
PD-1: Programmed cell death protein 1
LAG-3: Lymphocyte activation gene 3
IL-2: Interleukin 2
IL-10: Interleukin 10
TNF: Tumor necrosis factor
IFN-γ: Interferon-γ
FasL: Fas ligand
MFI: Median fluorescence intensity
SEM: Standard error of mean.
