# Supplementary figures and images for: The PI3K∂-Selective Inhibitor Idelalisib Induces T- and NK-Cell Dysfunction Independently of B-Cell Malignancy-Associated Immunosuppression
Source: Front Immunol. 2021 Mar 15;12:608625. doi: 10.3389/fimmu.2021.608625 (PMC8005712; doi:10.3389/fimmu.2021.608625)

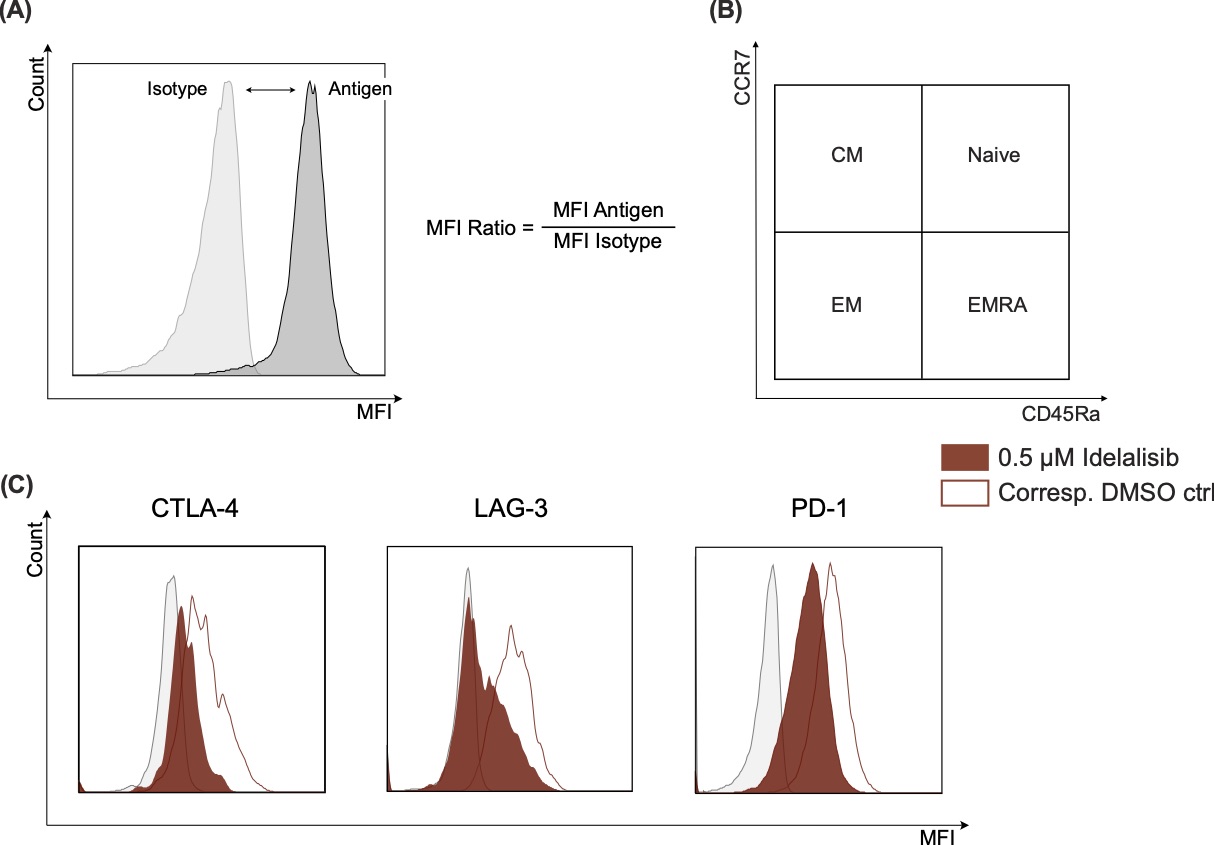

Supplement: Supplementary Figure 1 — Descriptive Plots. (A) Flow cytometry histogram: calculation of the MFI ratio (Median fluorescence intensity ratio). (B) Gating strategy T-cell subset discrimination. The four subsets were discriminated as follows: naïve T cells: CD45Ra+/CCR7+, memory T cells: CD45Ra−/CCR7+, effector T cells: CD45Ra−/CCR7−, and terminally differentiated effector memory T cells: CD45Ra+/CCR7−. (C) Representative flow cytometry histograms, MFI ratio: CTLA-4 (idelalisib = 1.9; DMSO = 4.7), LAG-3 (MFI ratios: idelalisib =2.3; DMSO = 19.5), and PD-1 (MFI-ratios: idelalisib = 13.7; DMSO = 39.3). [file Image_1.jpg]

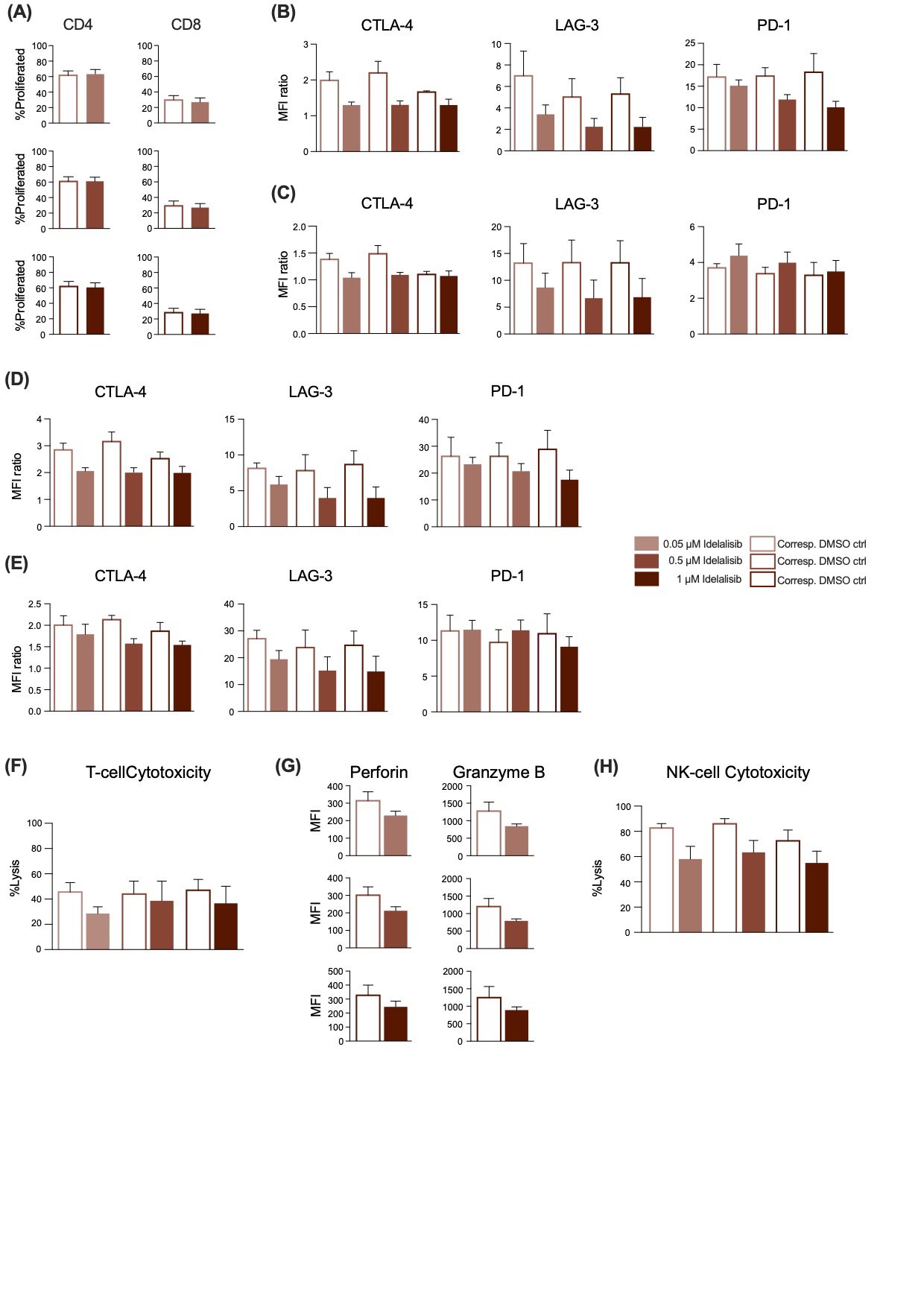

Supplement: Supplementary Figure 2 — Inhibition of PI3K∂ by idelalisib in an older reference cohort. (A) Compiled data showing the percentage of proliferated CD4+ and CD8+ T cells in the presence of various concentrations of idelalisib or DMSO. (B–E) Bar graphs of median fluorescence intensity (MFI) ratios showing the expression levels of checkpoint molecules CTLA-4, LAG-3, and PD-1 in CD4+ T cells, CD8+ T cells, CD4+ Tregs, and CD8+ Tregs after stimulation for 3 days with IL-2 and CD3/CD28 activation beads. (F) Cytolytic capacity of CD3+ T cells in coculture with HL-60 cell line and various concentrations of idelalisib after 72 h. (G) Bar charts depicting expression levels of perforin and granzyme B in CD8+ T cells after stimulation with CD3/CD28 beads for 72 h in the presence of various concentrations of idelalisib or DMSO. (H) Cytotoxicity of CD3−CD56+ NK cells against the K562 cell line in a flow cytometry-based coculture assay. Error bars represent ± SEM, n = 4 HDs in all depicted assays. [file Image_2.jpg]
