# Supplementary material for: The PI3K∂-Selective Inhibitor Idelalisib Induces T- and NK-Cell Dysfunction Independently of B-Cell Malignancy-Associated Immunosuppression
Source: Front Immunol. 2021 Mar 15;12:608625. doi: 10.3389/fimmu.2021.608625 (PMC8005712; doi:10.3389/fimmu.2021.608625)
Supplement: Supplementary Table 1 — Key Resources. [file Table_1.DOCX]

**Supplementary Table**

**Supplementary Table S1. Key Resources**

| **Antibodies** | | | | | |
| --- | --- | --- | --- | --- | --- |
| **Antibody** | **Source** | | **Identifier** | | **Catalog Number** |
| Antihuman CD2 | BioLegend (San Diego, CA, USA) | | TS1/8 | | 309218 |
| Antihuman CD3 | BioLegend (San Diego, CA, USA) | | UCHT1 | | 300434 |
| Antihuman CD3 | eBioscience (San Diego, CA, USA) | | SK7 | | 45-0036-42 |
| Antihuman CD4 | BD Biosciences (San Jose, CA, USA) | | RPA-T4 | | 560158 |
| Antihuman CD8 | BD Biosciences (San Jose, CA, USA) | | RPA-8 | | 555367 |
| Antihuman CD8 | BioLegend (San Diego, CA, USA) | | RPA-T8 | | 301032 |
| Antihuman CD16 | BioLegend (San Diego, CA, USA) | | 3G8 | | 302012 |
| Antihuman CD25 | BioLegend (San Diego, CA, USA) | | BC96 | | 302630 |
| Antihuman CD33 | eBioscience (San Diego, CA, USA) | | HIM3-4 | | 12-0339-42 |
| Antihuman CD45Ra | BioLegend (San Diego, CA, USA) | | HI100 | | 304130 |
| Antihuman CD56 | BioLegend (San Diego, CA, USA) | | HCD56 | | 318308 |
| Antihuman CD152 | BioLegend (San Diego, CA, USA) | | L3D10 | | 349906 |
| Antihuman CD178 | Miltenyi Biotec (Bergisch Gladbach,Germany) | | REA1056 | | 130-118-087 |
| Antihuman CD183 | BioLegend (San Diego, CA, USA) | | G025H7 | | 353720 |
| Antihuman CD197 | BioLegend (San Diego, CA, USA) | | G043H7 | | 353214 |
| Antihuman CD223 | eBioscience (San Diego, CA, USA) | | 3D5223H | | 25-2239-42 |
| Antihuman CD279 | BioLegend (San Diego, CA, USA) | | EH12.2H7 | | 329918 |
| Antihuman Granzyme B | BioLegend (San Diego, CA, USA) | | GB11 | | 515408 |
| Antihuman Perforin | BioLegend (San Diego, CA, USA) | | B-D48 | | 353311 |
| Antihuman TIGIT | eBioscience (San Diego, CA, USA) | | MBSA43 | | 11-9500-42 |
| Antihuman FoxP3 | eBioscience (San Diego, CA, USA) | | 236A/E7 | | 17-4777-42 |
| **Reagents and Kits** | | | | | |
| **Reagent** | | **Source** | | **Catalog Number** | |
| CellTrace CFSE | | Thermo Fisher Scientific (Waltham,MA, USA) | | C34554 | |
| LIVE/CEAD Fixable Aqua Dead Cell Stain Kit | | Thermo Fisher Scientific (Waltham,MA, USA) | | L34957 | |
| Calcein | | Thermo Fisher Scientific (Waltham,MA, USA) | | C3100MP | |
| Triton-X 100 | | Sigma Aldrich (Munich, Germany) | | X100-5ML | |
| pHrodo™ BioParticles® | | Thermo Fisher Scientific (Waltham,MA, USA) | | P35366 | |
| DMSO | | Serva (Heidelberg, Germany) | | 20385.01 | |
| Hepes | | Carl Roth (Karlsruhe, Germany) | | HN78.1 | |
| PSG | | Thermo Fisher Scientific (Waltham,MA, USA) | | 10378016 | |
| FCS | | Thermo Fisher Scientific (Waltham,MA, USA) | | 10270106 | |
| PBS | | Pan Biotech (Aidenbach, Germany) | | P04-36500 | |
| EDTA | | Thermo Fisher Scientific (Waltham,MA, USA) | | 15575-038 | |
| MACS BSA stock solution | | Miltenyi Biotec (Bergisch Gladbach,Germany) | | 130-091-376 | |
| CTS Dynabeads CD3/CD28 | | Thermo Fisher Scienific (Waltham,MA, USA) | | 40203D | |
| IL-2 | | R&D Systems (Minneapolis, MN, USA) | | 202-IL-010-CF | |
| RPMI 1640 | | Pan Biotech (Aidenbach, Germany) | | P04-16500 | |
| NK MACS Medium | | Miltenyi Biotec (Bergisch Gladbach,Germany) | | 130-114-429 | |
| EasySep Human T Cell Isolation Kit | | StemCell Technologies (Vancouver, BC, Canada) | | 17951 | |
| Pan T Cell Isolation Kit | | Miltenyi Biotec (Bergisch Gladbach,Germany) | | 130-096-535 | |
| EasySep Human CD56 Positive Selection Kit | | StemCell Technologies (Vancouver, BC, Canada) | | 17815 | |
| EasySep Direct Neutrophil Isolation Kit | | StemCell Technologies (Vancouver, BC, Canada) | | 19666 | |
| NK Cell Isolation Kit, human | | Miltenyi Biotec (Bergisch Gladbach,Germany) | | 130-092-657 | |
| CBA Human Th1/Th2 Cytokine Kit (RUO) | | BD Biosciences (San Jose, CA, USA) | | 551809 | |
